# The Association between Adult Weight Gain and Insulin Resistance at Middle Age: Mediation by Visceral Fat and Liver Fat

**DOI:** 10.3390/jcm8101559

**Published:** 2019-09-28

**Authors:** Inge Verkouter, Raymond Noordam, Saskia le Cessie, Rob M. van Dam, Hildo J. Lamb, Frits R. Rosendaal, Diana van Heemst, Renée de Mutsert

**Affiliations:** 1 Department of Clinical Epidemiology, Leiden University Medical Center, 2333 ZA Leiden, The Netherlands; i.verkouter@lumc.nl (I.V.);; 2 Department of Internal Medicine, Section Gerontology and Geriatrics, Leiden University Medical Center, 2333 ZA Leiden, The Netherlands; 3 Department of Biomedical Data Sciences, Leiden University Medical Center, 2333 ZA Leiden, The Netherlands; 4 Saw Swee Hock School of Public Health and Department of Medicine, Yong Loo Lin School of Medicine, National University of Singapore and National University Health System, Singapore 117549, Singapore; 5 Department of Radiology, Leiden University Medical Center, 2333 ZA Leiden, The Netherlands

**Keywords:** body weight changes, visceral fat, fatty liver, insulin resistance

## Abstract

We aimed to investigate the role of the amount of visceral fat and liver fat in the association between adult weight change and insulin resistance at middle age. In the Netherlands Epidemiology of Obesity study, adult weight change was calculated with recalled body weight at age 20 years and measured body weight at middle age. Measures of insulin resistance were calculated using both fasting and postprandial glucose and insulin concentrations. Visceral fat was assessed by magnetic resonance (MR) imaging and liver fat by proton-MR spectroscopy (*N* = 1758). We examined the association between adult weight change and insulin resistance with linear regression, adjusted for confounding factors. To investigate mediation, we additionally adjusted for total body fat, visceral fat, and liver fat. In participants who gained ≥50% of body weight during adulthood, homeostatic model assessment for insulin resistance (HOMA-IR) was 3.22 (95% CI 2.76; 3.77) times higher than in weight maintainers. In a joint model, total body fat mediated this association for 8.1% (95% CI −9.2; 25.4), visceral fat for 32.0% (18.6; 45.4%) and liver fat for 22.5% (15.0; 30.1). The association between adult weight gain and insulin resistance at middle age is largely mediated by both visceral fat and liver fat.

## 1. Introduction

Adult weight gain and obesity are well-established causal risk factors for the development of type 2 diabetes mellitus, cardiovascular disease and obesity-related cancers [1,2,3]. In line with these findings, adult weight gain was strongly associated with increased insulin resistance in multiple studies [4,5,6,7,8,9].

It is well-established that abdominal adiposity, and in particular visceral adipose tissue, is strongly related to insulin resistance and risk of type 2 diabetes mellitus, also after adjustment for total body fat [10,11,12,13,14,15]. In a previous analysis, we observed that larger gain in body weight during adulthood was associated with more visceral fat and liver fat at middle age, compared with weight maintenance [16]. This finding is in agreement with the ‘lipid overflow’ hypothesis, which postulates that lipids are stored in the visceral area and in and around organs (ectopic fat) when the subcutaneous adipose tissue has reached its limited storage capacity [17,18]. In addition to visceral adipose tissue, excess liver fat has been associated with insulin resistance [19], as well as with a higher risk of type 2 diabetes mellitus, cardiovascular disease and mortality [20].

As both excess visceral fat and liver fat are strongly associated with increased insulin resistance and type 2 diabetes, we hypothesized that the association between adult weight change and insulin resistance is largely mediated by both excess visceral fat and liver fat. Therefore, the aim of this study was to investigate to what extent the association of adult weight change with insulin resistance is mediated by the amounts of visceral fat and liver fat at middle age.

## 2. Methods

### 2.1. Study Design and Study Population

The Netherlands Epidemiology of Obesity (NEO) study is a population-based cohort study of 6671 individuals aged 45–65 years, with an oversampling of individuals with body mass index (BMI) ≥ 27 kg/m^2^, living in the greater area of Leiden (in the West of the Netherlands). All inhabitants aged between 45 and 65 years from one municipality (Leiderdorp) were invited to participate irrespective of their BMI, allowing for a reference distribution of BMI. The study design and population are described in detail elsewhere [21].

Participants were invited to a baseline visit at the NEO study center of the Leiden University Medical Center (LUMC) after an overnight fast. Prior to the study visit, participants completed a general questionnaire at home to report demographic, lifestyle and clinical information. At the study center, participants completed a screening form, asking about anything that might create a health risk or interfere with magnetic resonance imaging, e.g., presence of metallic devices, claustrophobia and a body circumference > 1.70 m. Of the eligible participants, 2580 participants were randomly selected to undergo magnetic resonance imaging(MRI) [21]. This subset of participants from the NEO cohort has similar characteristics as the participants from the NEO cohort not participating in this subset, apart from a slightly higher BMI and more individuals with a history of cardiovascular disease in the subset that did not participate [15]. The Medical Ethical Committee of the LUMC approved the NEO study. All participants provided written informed consent.

For the present study, we performed cross-sectional analyses with baseline measurements of the NEO study. Hepatic triglyceride content was available in 2086 of the participants who underwent MRI, due to technical failures or an insufficient quality of the measurements to estimate liver fat content. We excluded participants with images of insufficient quality to estimate visceral adipose tissue (*n* = 11), with missing data on recalled body weight at age 20 years (*n* = 60), and with a BMI at age 20 below 14.0 kg/m^2^ (*n* = 1). Additionally, we excluded participants who used glucose-lowering medication (*n* = 129), were non-fasting at baseline (*n* = 1), or had missing data on postprandial glucose at 30 min (*n* = 38), at 150 min (*n* = 30) or fasting insulin (*n* = 3), total body fat at baseline (*n* = 3), ethnicity (*n* = 2), educational level (*n* = 15) and physical activity (*n* = 35), resulting in 1758 participants (913 men, BMI range 20.1–39.6 kg/m^2^ and 845 women, BMI range 18.2–45.3 kg/m^2^) who were included in the analyses. The majority of our study population was of Caucasian ethnicity (96.3%). Other ethnicities that were represented in our study sample are African (0.5%), Turkish (0.3%), South-East Asian (0.5%), Hindu (0.1%), and ethnicities other than aforementioned (2.3%).

### 2.2. Data Collection

#### 2.2.1. Weight Change during Adulthood

At the baseline study visit, height without shoes was measured with a vertically fixed, calibrated tape measure. Body weight was measured and percent body fat was estimated by the Tanita bio impedance balance (TBF-310, Tanita International Division, UK) without shoes and 1 kg was subtracted to correct for weight of clothing. BMI at baseline was calculated by dividing the weight in kg by the height in meters squared.

Recalled weight at the age of 20 years was based on self-report. The general questionnaire included the question ‘How much did you weigh (approximately) when you were 20 years old?’. BMI at age 20 years was calculated by dividing body weight at age 20 in kg by the height in meters squared at middle age with the assumption that height did not majorly change during adulthood. Relative weight change was calculated by subtracting weight at age 20 from measured baseline weight, divided by weight at age 20, and multiplied by 100% [16].

#### 2.2.2. Visceral Fat and Liver Fat at Middle Age

Visceral adipose tissue was directly assessed by MRI (1.5 Tesla MR imaging, Philips Medical Systems, Best, Netherlands) using a turbo spin echo imaging protocol with the following imaging parameters: 300/20; flip angle, 90°; section thickness, 10 mm, section gap, 2 mm. At the level of the fifth lumbar vertebra, three transverse slices were obtained during one breath-hold [21]. Imaging parameters were: TR = 300 ms; TE = 20ms; flip angle = 90°; slice thickness = 10 mm, slice gap = 2 mm. Visceral fat areas were quantified by converting the number of pixels to centimeters squared for all three slices and totaling the areas of the three sections, using in-house-developed software (MASS; Leiden University Medical Center, Leiden, the Netherlands).

Hepatic triglyceride content was quantified using ^1^H-magnetic resonance spectroscopy of the liver [21]. An 8 mL voxel was positioned in the right lobe of the liver, avoiding gross vascular structures and adipose tissue depots. Sixty-four averages were collected with water suppression (repetition time = 2900 ms; echo time = 23 ms (2900/23). Data points (1024) were collected by using a 1000-Hz spectral line. Without changing any parameters, spectra without water suppression, with a repetition time of 10 s and with four averages were obtained as internal reference. Hepatic triglyceride content relative to water was calculated as (signal amplitude of triglyceride)/(signal amplitude of water) × 100. Spectra were not corrected for frequency drift. Spectral data were analyzed while blinded to all study parameters, including age, sex, LV function and dimensions, BMI, waist circumference, visceral adipose tissue, and total body fat. Spectra were initially included when automatic fitting was successful. When line shapes were distorted by eddy currents or as a result of poor shimming, spectral data were rejected.

#### 2.2.3. Measures of Insulin Resistance at Middle Age

Fasting blood samples were drawn from the antecubal vein after 5 min rest of the participant, after an overnight fast of at least 10 h. Within 5 min after drawing a fasting blood sample, all participants consumed a liquid mixed meal (400 mL) that contained 2.5 megajoule (MJ), of which 16 percent of energy (En%) was derived from protein, 50 En% from carbohydrates and 34 En% from fat. Subsequently, blood samples were drawn after 30 and 150 min. Plasma glucose concentrations were determined by enzymatic and colorimetric methods (Roche Modular Analytics P800, Roche Diagnostics, Mannheim, Germany; CV < 5%) and serum insulin concentrations were determined by an immunometric method (Siemens Immulite 2500, Siemens Healthcare Diagnostics, Breda, The Netherlands; CV < 5%) at the Department of Clinical Chemistry and Laboratory Medicine of the LUMC [21].

From fasting glucose and insulin concentrations, we calculated the Homeostasis Model Assessment for Insulin Resistance (HOMA-IR), a marker of hepatic insulin resistance [22]. HOMA-IR was calculated as fasting insulin (μU/mL) × fasting glucose (mmol/L)/22.5 [22,23]. Matsuda Insulin Sensitivity Index (ISI) was calculated as 10,000/square root (fasting glucose (mg/dL) × fasting insulin (μU/mL)) × (mean_glucose0-_150 × mean_insulin0-150_) [24,25,26].

#### 2.2.4. Covariates

Ethnicity was self-identified in the questionnaire and regrouped into Caucasian and other. Highest level of education was reported in ten categories according to the Dutch education system and regrouped in two categories: Low education (no education, primary school or lower vocational education) and high education (higher vocational education, university and postgraduate education). Smoking status was self-reported. Alcohol consumption was reported on the food frequency questionnaire and expressed as grams/day [27]. Participants reported the frequency and duration of their usual physical activity during leisure time in the Short questionnaire to assess health-enhancing physical activity (SQUASH), which was expressed in hours per week of metabolic equivalents [28,29]. Family history of diabetes mellitus and myocardial infarction were reported as having any parent or sibling with diabetes mellitus or not (reference group).

### 2.3. Statistical Analyses

In the NEO study, individuals with BMI ≥ 27 kg/m^2^ are oversampled. To correctly represent associations for the general population [30], adjustments for the oversampling were made. This was done by weighting all participants towards the BMI distribution of participants from the Leiderdorp municipality [31], whose BMI distribution was similar to the BMI distribution of the general Dutch population [32]. All results were based on weighted analyses and are therefore generalizable to a population-based study without oversampling of individuals with BMI ≥ 27 kg/m^2^ [21]. As a consequence of the weighting procedure, numbers of participants per category are presented as percentages.

Characteristics of the study population at middle age were expressed as mean (SD), median (25th, 27th percentiles or range) or as percentage, stratified by categories of weight change. We explored the distribution of weight change in the reference population of Leiderdorp in an earlier study, and observed that the majority of the population gained weight between age 20 years and middle age in this era [16]. Therefore, we decided to stratify categories of adult weight change as: weight loss of more than 5%, weight change between −5% and 5% (weight maintenance: reference category), weight gain of 5% to 25%, 25% to 50%, and ≥50%.

We performed linear regression analyses to examine the associations of adult weight change with fasting and postprandial glucose and insulin concentrations and with measures of insulin resistance at middle age, compared with the reference category of weight maintenance during adulthood. Potential confounding factors were defined a priori based on biological knowledge from previous studies. Crude models were adjusted for sex and age (model 1). In model 2, we additionally adjusted for BMI at age 20, because the percentage of weight change since age 20 depends on initial BMI at age 20. In model 3, we additionally adjusted for ethnicity, education, smoking, alcohol consumption, physical activity, and family history of diabetes. Because of a skewed distribution, values of fasting and postprandial glucose and insulin, HOMA-IR and Matsuda ISI were all transformed to the natural logarithm. Regression coefficients and corresponding 95% confidence intervals (95% CI) were back transformed and expressed as ratios, which can be interpreted as the relative changes in insulin resistance in a certain weight change category, compared with insulin resistance in the reference category of weight maintenance. For example: a ratio of 2 for HOMA-IR in individuals who gained 5% to 25% of body weight during adulthood indicates that these individuals have twofold higher HOMA-IR values at middle age than individuals who maintained their body weight during adulthood.

Subsequently, we examined mediation in the association between adult weight change and insulin resistance at middle age by total body fat, visceral fat, and liver fat at middle age according to the method proposed by Baron and Kenny [33]. This method is based on comparing the regression coefficient of the association between an exposure and outcome, and the regression coefficient of the association between the exposure and outcome adjusted for the mediating variable. First, we checked whether the exposure–outcome, exposure–mediator, and mediator–outcome associations were present in our study. Associations between adult weight change and total body fat, visceral fat, and liver fat were previously described for the NEO population [16], as well as the associations between total body fat, visceral fat, and liver fat and insulin resistance (HOMA-IR) [15]. The association between adult weight change and insulin resistance was examined in the present study. Secondly, we checked the assumption of no interaction between the exposure and mediator by examining the interaction between adult weight change and total body fat, visceral adipose tissue and hepatic triglyceride content in the association with insulin resistance. We attempted to avoid mediator-outcome confounding in the mediation analyses by adjusting for measured potential confounding factors.

Additional to Baron and Kenny’s mediation method, we used structural equation modelling (SEM) to evaluate the effect of several mediators on the relation between adult weight change and insulin resistance adjusted for possible confounding factors, expressed as a percentage of mediation [34]. By multiplying the regression coefficients of the exposure-mediator and the mediator-outcome model, we were able to calculate the separate and combined indirect effects of total body fat, visceral fat and liver fat on the association between adult weight change and insulin resistance, with their corresponding 95% CIs. We divided the indirect effects by the total effect, which is the sum of the direct and indirect effects, to calculate the percentage of mediation by the different mediators and corresponding 95% CI.

We also conducted several sensitivity analyses. We repeated all analyses with absolute adult weight change between age 20 years and middle age in kilograms, instead of the relative weight change as a percentage. We repeated the analyses with relative weight change as a continuous variable. Additionally, we assessed whether there was interaction by sex in the association between adult weight change and insulin resistance by including a product term of sex and adult weight change, and repeated all analyses for men and women separately.

## 3. Results

### 3.1. Characteristics of the Study Population

A total of 1758 individuals (54% women) were analyzed in the present study. Mean (SD) age of the study population was 55 (6) years, mean BMI at age 20 years was 21.8 (2.6) kg/m^2^, mean BMI at middle age was 25.8 (3.9) kg/m^2^, and mean percentage of adult weight change was a gain in weight of 19.1% (16.0%).

Characteristics of the study population stratified by five weight change categories are presented in Table 1. The proportion of women was higher in participants who gained more than 25% of body weight between age 20 and middle age than in participants who gained less than 25% during adulthood. Additionally, participants who gained more than 25% of body weight were less likely to be highly educated, and waist circumference, total body fat, visceral fat and liver fat at middle age were higher than in participants who gained less than 25% of body weight, in both men and women. Median fasting plasma glucose, fasting serum insulin and HOMA-IR were higher in individuals who gained more than 25% of body weight, compared with individuals who gained less than 25% of body weight, whereas Matsuda ISI was lower.

### 3.2. Adult Weight Change and Insulin Resistance at Middle Age

**Figure 1:**
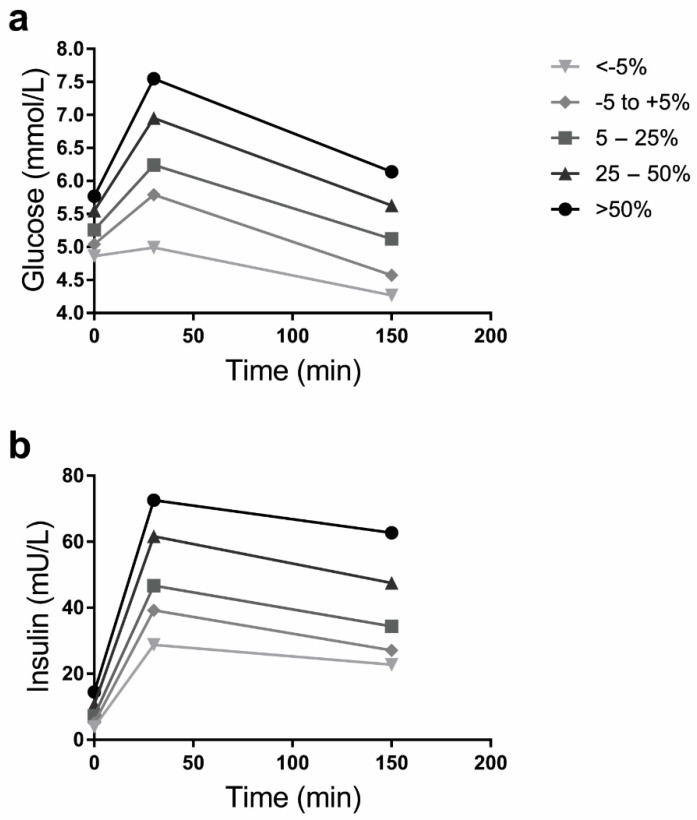
Estimated means of (**a**) glucose (mmol/L) and (**b**) insulin (mU/L) blood concentrations fasting, and at *t* = 30 and *t* = 150 min after a mixed meal challenge, stratified by adult weight change (*N* = 1758) and adjusted for sex, age, BMI at age 20, ethnicity, education, smoking, alcohol consumption, physical activity, and family history of diabetes.

As shown in Figure 1, each higher category of change in body weight during adulthood was associated with higher fasting and postprandial glucose and insulin concentrations at middle age, after adjustment for sex, age, BMI at age 20, ethnicity, education, smoking, alcohol consumption, physical activity, and family history of diabetes.

After adjustment for potential confounding factors (model 2, Table 2), HOMA-IR was 1.37 (95% CI 1.20; 1.56) times higher in participants who gained 5–25% of body weight, 2.04 (1.79; 2.34) times higher in participants who gained 25–50% of body weight, and 2.65 (2.24; 3.14) times higher in those who gained ≥50% of body weight during adulthood than in weight maintainers. The ratio of relative change in the Matsuda ISI was 0.76 (95% CI 0.68; 0.84) in participants who gained 5–25% of body weight during adulthood, 0.51 (0.46; 0.58) in participants who gained 25–50% of body weight compared with weight maintainers, and 0.40 (0.34; 0.47) in participants who gained ≥50% of body weight in model 2. After additional adjustment for BMI at age 20 (model 3), associations between adult weight change and both HOMA-IR and Matsuda ISI became slightly stronger (Table 2). Adjustment for sex, age and BMI at age 20 had the largest impact on the associations between adult weight gain and insulin resistance.

When using absolute adult weight change (in kg) instead of relative weight change, results were similar (Table S1). Additionally, we repeated the analyses with adult weight change as continuous variable (per 10% of weight change, Table S2), showing similar results.

### 3.3. Mediation Analyses

We did not observe interaction between the mediators (total body fat, visceral adipose tissue and hepatic triglyceride content) and adult weight change in the association with HOMA-IR or Matsuda ISI (Table S3).

**Figure 2:**
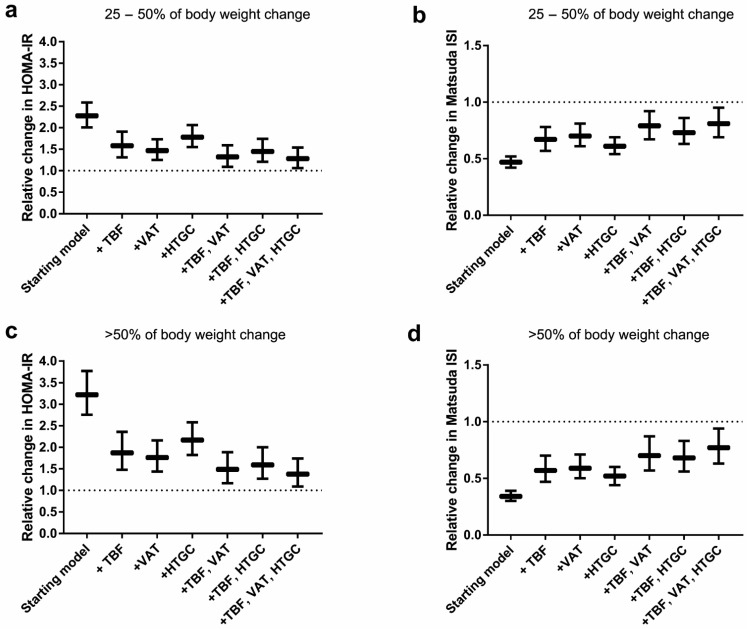
Relative changes (95% CI) of measures of HOMA-IR and Matsuda Index for participants who gained 25–50% of body weight (**a,b**) or >50% of body weight (**c,d**) after addition of mediators, compared to weight maintainers. TBF, total body fat; VAT, visceral adipose tissue; HTGC, hepatic triglyceride content. Starting model (model 3) was adjusted for sex, age, BMI at age 20, ethnicity, education, smoking, alcohol consumption, physical activity and family history of diabetes.

As presented in Figure 2 and Table S4, after adjustment for total body fat at middle age, the association between adult weight change and HOMA-IR attenuated across all weight change categories. For example, individuals who gained ≥50% of body weight during adulthood had 1.87 (1.48; 2.36) times higher mean HOMA-IR values. After additional adjustment for visceral fat, the association attenuated further to 1.49 (1.17; 1.89), while after adjustment for liver fat to 1.59 (1.27; 2.00). When we adjusted the association between adult weight change and HOMA-IR for total body fat, visceral fat and liver fat in one model, the association was 1.38 (1.09; 1.74) for individuals who gained ≥50% of body weight. For Matsuda ISI, the pattern of associations was in opposite direction, but similar to HOMA-IR (Figure 2 and Table S4).

Results for sex-stratified mediation analyses are presented in Table S5 (men) and Table S6 (women). We observed similar patterns of mediation by visceral fat and liver fat in men and women. Additionally, we did not observe interaction by sex in the association between adult weight change and HOMA-IR or Matsuda ISI (*p*-value for interaction 0.53 and 0.67, respectively).

In addition, we also performed SEM analyses to estimate the indirect effects of adult weight change through total body fat, visceral fat and liver fat as a percentage of its total effect (Table 3). Separately, total body fat, visceral adipose tissue and hepatic triglyceride content all had an indirect effect in the association between adult weight change and HOMA-IR (Table 3). However, when the joint mediating effect of total body fat and visceral fat or liver fat was considered, the indirect effect via total body fat disappeared in women, but remained in men. When all three mediators were included in the model, the percentage of mediation of the total association between adult weight change and HOMA-IR was 32.0% (95% CI 18.6; 45.4) for visceral fat and 22.5% (15.0; 30.1) for liver fat. Similar percentages of mediation were observed for Matsuda ISI (results not shown).

## 4. Discussion

The aim of our study was to investigate the association between adult weight change and insulin resistance at middle age, and to what extent this association is mediated by visceral fat and liver fat. In this population-based study of 1758 men and women, we observed that a gain in body weight during adulthood as small as 5% was already associated with more insulin resistance compared with weight maintenance during adulthood. Stronger associations with insulin resistance were observed for more excessive weight gain during adulthood. When considering the combined mediation effect, by adjusting for total body fat, visceral fat and liver fat in one model, we observed that the association between adult weight change and insulin resistance was 8.1% mediated by total body fat, 32.0% by visceral fat and 22.5% by liver fat. After adjustment for total body fat, visceral fat or liver fat separately, we observed the largest attenuation of the association between adult weight change and insulin resistance after adjustment for total body fat or visceral fat, compared with adjustment for liver fat. However, this results can be explained by the strong correlation between total body fat, visceral fat and liver fat, and thereby their overlapping mediation roles.

After adjustment for total body fat, visceral fat and liver fat at middle age, insulin resistance at middle age of individuals who had gained more than 50% of body weight was still 1.38-fold higher than the insulin resistance of weight maintainers. This difference can be explained by either residual confounding or measurement error in the assessment of total body fat, because total body fat was estimated by bioelectrical impedance analysis (BIA). The estimation of total body fat percentage by BIA showed good absolute agreement (intraclass correlation coefficient 0.90, 95% CI 0.89; 0.91) with total body fat percentage by dual-energy X-ray absorptiometry (DXA) which was available in a small subset of the NEO study (*N* = 915). Alternatively, the remainder of the association between weight change and insulin resistance might be mediated by ectopic fat deposition in organs other than the liver, such as the heart, pancreas, kidneys, and skeletal muscles [18]. Because this information is not available in the NEO study, we were unable to investigate this hypothesis.

A meta-analysis based on 15 observational studies showed that the risk of type 2 diabetes mellitus was increasing in line with an increasing gain in body weight during adulthood, suggesting a dose-response association [35], in agreement with the results of our study. Also in line with our results, in a cross-sectional study of 153 middle-aged women HOMA-IR was higher in women who had gained more than 30 kg of body weight since the age of 20 than in women who had gained less than 10 kg [9]. A study in Japanese adults (*N* = 399) also showed that weight gain since age 20 years was associated with higher HOMA-IR [36]. The authors noted that this association attenuated after adjustment for BMI at middle age, suggesting that the association between weight gain and HOMA-IR was largely explained by the participants’ BMI at middle age. In our study, we adjusted for total body fat, visceral fat and liver fat and showed that the association between adult weight change and insulin resistance is mostly mediated by visceral fat and liver fat.

The biological mechanism underlying these observations could be a reflection of the eventual limited capacity of subcutaneous adipose tissue to store lipids during weight gain [14,18]. When the capacity threshold of adipose tissue is reached, lipids will be stored in the visceral area and subsequently will be deposited in ectopic sites such as the liver, heart, muscles and pancreas [18,37]. Here, the visceral fat cells will exert their detrimental effects by secreting cytokines and non-esterified fatty acids (NEFAs) or very low-density lipoproteins [14,38,39,40,41]. Elevation in release of pro-inflammatory cytokines such as IL-6 and TNF-α may induce a low-grade inflammatory state and oxidative stress, eventually leading to insulin resistance. Intracellular NEFAs inhibit insulin signaling, which will also lead to insulin resistance [42].

Strengths of our study include the large study population, data on many potential confounding factors, and the availability of directly assessed visceral adipose tissue by MRI and hepatic triglyceride content by ^1^H-MRS, providing more accurate measures of abdominal adiposity than waist circumference. This enabled us to assess the mediating effects of visceral fat and liver fat after taking mediation via total body fat into account.

A limitation that needs to be considered is assessment of insulin resistance by HOMA-IR and Matsuda ISI. The golden standard measurement of insulin resistance is the hyperinsulinemic euglycemic clamp [43]. However, this is not feasible in a large study population. Instead, we calculated HOMA-IR and Matsuda ISI based on both fasting and postprandial glucose and insulin concentrations, which are valid surrogate measures for insulin resistance in large population-based studies [44]. The fact that the results of these two different proxies of insulin resistance were similar, suggests that our findings are robust. Second, we calculated BMI at age 20 using recalled weight at age 20. Therefore, body weight at age 20 and weight change during adulthood might have been misclassified. However, previous studies have shown that recalled weight is highly correlated with measured weight at the same age [45], and in a previous study we showed that the association between adult weight change and measures of (abdominal) adiposity did not markedly change after correction for measurement error in recalled weight at age 20 years [16]. Third, the majority of our study population was Caucasian, therefore the results of our study need to be confirmed in other ethnic groups.

In conclusion, our results indicate that the association between adult weight gain and insulin resistance at middle age is largely mediated by visceral fat and liver fat at middle age, which is increasingly important given the growing prevalence of abdominal obesity and non-alcoholic fatty liver disease [46,47]. Our results suggest that weight maintenance during adulthood plays an important role in preventing accumulation of excess visceral fat and liver fat and thereby insulin resistance and eventually, type 2 diabetes at middle age and older age. Future prospective studies need to investigate the precise mechanisms by which visceral fat and liver fat lead to insulin resistance, and ways to reduce or prevent visceral fat and liver fat accumulation.

## Figures and Tables

**Table 1:** Characteristics of participants of the Netherlands Epidemiology of Obesity (NEO) study, aged 45 to 65 years, with measurements of visceral adipose tissue and hepatic triglyceride content by magnetic resonance imaging and spectroscopy, stratified by adult weight change (*N* = 1758).

	Loss of >5%	Weight Maintenance −5% to <5%	Gain of ≥5% to <25%	Gain of ≥25% to <50%	Gain of ≥50%
Proportion of population (%)	4.5	11.2	55.0	24.7	4.6
Sex (% men)	22	39	50	46	38
Body weight at age 20					
Recalled weight at age 20 (kg)	73.7 (8.0)	67.2 (7.6)	66.2 (9.3)	64.5 (14.1)	59.0 (14.5)
BMI at age 20 (kg/m^2^)	25.1 (2.1)	22.6 (1.9)	21.7 (1.9)	21.3 (3.3)	19.9 (4.1)
Change in weight (%, range)	−7.6 (−32.2; −5.8)	1.9 (−4.9; 4.8)	14.9 (5.0; 24.9)	32.3(25.0; 49.8)	57.2 (50.0;102.8)
Characteristics at middle age					
Age (years)	53 (3)	57 (4)	55 (5)	55 (7)	56 (8)
Ethnicity (% Caucasian)	100	96	96	98	91
Education (% high)	45	53	51	37	31
Smoking (% current)	19	21	13	13	11
Alcohol (g/day)	4 (1–21)	10 (4–16)	11 (3–23)	8 (2–21)	8 (1–21)
Physical activity (MET-hours/week)	27 (19–58)	42 (28–56)	31 (17–53)	26 (14–44)	20 (10–42)
Body weight (kg)	66.0 (6.2)	67.9 (7.8)	76.0 (11.2)	86.3 (18.7)	94.6 (23.3)
BMI (kg/m^2^)	22.4 (1.4)	22.8 (1.9)	25.0 (2.4)	28.5 (4.6)	31.9 (6.6)
Waist circumference (cm, M/W)	90(5)/76(5)	87(6)/77(7)	96(7)/82(7)	105(12)/93(13)	110(14) /103(16)
Total body fat (%, M/W)	18(3)/32(3)	20(3)/31(4)	24(3)/35(4)	28(7)/41(6)	31(8)/44(10)
Visceral adipose tissue (cm^2^, M/W)	50(44–66)/21(14–37)	50(19–79)/36(24–47)	98(76–133)/49(35–69)	135(104–173)/88(59–113)	158(131–210)/118(94–156)
Hepatic triglyceride content (%, M/W)	2.2(0.9–2.6)/0.9(0.7–1.6)	1.8(1.0–3.6)/1.2(0.7–1.7)	3.5(2.0–7.0)/1.6(1.1–3.6)	6.0(3.5–14.0)/3.4(1.6–8.4)	11.8(3.8–20.8)/7.7(3.7–18.8)
In women ^a^:					
Postmenopausal (% yes)	37	78	51	66	69
Current use of sex hormones ^b^ (%)	4	3	11	7	3
Insulin resistance at middle age					
Family history of diabetes (% yes)	31	22	26	24	31
Family history of myocardial infarction (% yes)	26	34	39	47	48
Fasted plasma glucose (mmol/L)	4.8 (4.5–5.1)	5.1 (4.8–5.3)	5.2 (4.9–5.6)	5.5 (5.2–5.9)	5.6 (5.3–6.1)
Fasted serum insulin (mU/L)	5.5 (4.1–6.5)	5.4 (3.6–7.1)	7.3 (5.2–9.9)	10.5 (7.6–14.7)	13.0 (8.6–21.6)
HOMA-IR	1.1 (0.8–1.5)	1.2 (0.8–1.6)	1.7 (1.2–2.4)	2.6 (1.8–3.7)	3.2 (2.1–5.5)
Matsuda ISI	2.4 (2.1–2.7)	2.1 (1.8–2.5)	1.8 (1.5–2.1)	1.4 (1.0–1.8)	1.1 (0.6–1.6)

^a^ 54% of study population. ^b^ Use of sex hormones included oral contraceptive and hormonal replacement therapy. Results were based on analyses weighted towards the BMI distribution of the general population (*N* = 1758). Abbreviations: BMI, body mass index; MET, metabolic equivalent of task; M, men; W, women; HOMA-IR, homeostatic model assessment insulin resistance; Matsuda ISI, Matsuda insulin sensitivity index. Data are presented as mean (SD), median (25th–75th percentile/range) or percentage.

**Table 2:** Relative change with 95% confidence intervals in measures of insulin resistance and insulin sensitivity for categories of weight change during adulthood, compared with weight maintenance (*N* = 1758).

	Model 1		Model 2		Model 3	
	Ratio	95% CI	Ratio	95% CI	Ratio	95% CI
HOMA-IR						
< −5.0%	0.85	0.56; 1.30	0.85	0.56; 1.30	0.73	0.47; 1.12
−5% to 5% (ref)	1		1		1	
5–25%	1.38	1.21; 1.57	1.37	1.20; 1.56	1.47	1.30; 1.67
25–50%	2.14	1.87; 2.44	2.04	1.79; 2.34	2.28	2.01; 2.59
>50%	2.78	2.34; 3.30	2.65	2.24; 3.14	3.22	2.76; 3.77
Matsuda ISI						
< −5.0%	1.22	0.92; 1.63	1.23	0.92; 1.63	1.40	1.05; 1.86
−5 to 5% (ref)	1		1		1	
5–25%	0.75	0.67; 0.84	0.76	0.68; 0.84	0.71	0.64; 0.79
25–50%	0.49	0.44; 0.55	0.51	0.46; 0.58	0.47	0.42; 0.52
>50%	0.38	0.32; 0.44	0.40	0.34; 0.47	0.34	0.30; 0.39

Results were based on analyses weighted towards the BMI distribution of the general population and were derived from beta coefficients with 95% confidence intervals from linear regression analyses and expressed as ratios of outcome measures compared with weight maintenance during adulthood. Abbreviations: CI, confidence interval; HOMA-IR, homeostatic model assessment insulin resistance; Matsuda ISI, Matsuda insulin sensitivity index; ref, reference group. Model 1: Adjusted for sex and age; 2: additionally adjusted for ethnicity, education, smoking, alcohol consumption, physical activity and family history of diabetes; 3: additionally adjusted for BMI at age 20.

**Table 3:** Analysis of indirect effects of the mediators total body fat, visceral adipose tissue and hepatic triglyceride content in the association between adult weight gain and HOMA-IR.

		All *(N = 1758)*		Men *(N = 913)*		Women *(N = 845)*	
		% of Total Effect	95% CI	% of Total Effect	95% CI	% of Total Effect	95% CI
Total effect		100		100		100	
Indirect effect through:							
TBF alone		34.2	16.6; 51.9	42.2	20.6; 63.9	27.3	−0.4; 55.0
VAT alone		44.1	31.3; 56.9	31.9	19.3; 44.6	51.2	29.6; 72.8
HTGC alone		28.3	20.9; 35.8	25.8	14.9; 36.8	29.1	19.1; 39.1
TBF + VAT	TBF	13.0	−4.4; 30.3	29.8	9.0; 50.6	−1.7	−0.29.9; 26.5
	VAT	41.6	28.7; 54.4	28.5	16.1; 41.0	51.7	29.4; 73.9
TBF + HTGC	TBF	22.5	4.5; 40.5	28.6	7.6; 49.7	16.6	−12.3; 45.5
	HTGC	27.0	19.4; 34.6	24.2	13.4; 35.0	28.1	17.4; 38.8
TBF + VAT + HTGC	TBF	8.1	−9.2; 25.4	20.2	−0.4; 40.9	−3.2	−30.8; 24.4
	VAT	32.0	18.6; 45.4	22.5	10.2; 34.7	39.5	15.5; 63.4
	HTGC	22.5	15.0; 30.1	21.8	11.6; 32.0	21.9	10.6; 33.3

Results were based on analyses weighted towards the BMI distribution of the general population and were derived from multiplied path coefficients with 95% confidence intervals from structural equation modelling (path analysis) and expressed as indirect effects in the association between adult weight change (per 10% weight change) and insulin resistance. Indirect effects were divided by total effects to calculate the percentage mediated. Abbreviations: CI, confidence interval; HOMA-IR, homeostatic model assessment insulin resistance; TBF, total body fat; VAT, visceral adipose tissue; HTGC, hepatic triglyceride content. Indirect effects were adjusted for sex, age, BMI at age 20, ethnicity, education, smoking, alcohol consumption, physical activity and family history of diabetes.

## Supplementary Materials

The following are available online at https://www.mdpi.com/2077-0383/8/10/1559/s1, Table S1: Relative change with 95% confidence intervals in measures of insulin resistance and sensitivity for categories of absolute weight change (in kilograms) during adulthood, compared with weight maintenance (*N* = 1758), Table S2: Association between of adult weight change (per 10% change) and measures of insulin resistance and sensitivity, for complete study population and for men and women separately, Table S3: P-values for interaction between total body fat, visceral adipose tissue and hepatic triglyceride content and adult weight change in the association with measures of insulin resistance (*N* = 1758), Table S4: Mediation of the association between adult weight change and insulin resistance by total body fat, visceral adipose tissue and hepatic triglyceride content (*N* = 1758), Table S5: Mediation of the association between adult weight change and insulin resistance by total body fat, visceral adipose tissue and hepatic triglyceride content in men (*N* = 913), Table S6: Mediation of the association between adult weight change and insulin resistance by total body fat, visceral adipose tissue and hepatic triglyceride content in women (*N* = 845)
