# Supplementary material for: The Association between Adult Weight Gain and Insulin Resistance at Middle Age: Mediation by Visceral Fat and Liver Fat
Source: J Clin Med. 2019 Sep 28;8(10):1559. doi: 10.3390/jcm8101559 (PMC6832997; doi:10.3390/jcm8101559)
Supplement: Supplementary file 1 [file jcm-08-01559-s001.pdf]

Table S1. Relative change with 95% confidence intervals in measures of insulin resistance and sensitivity for categories of absolute weight change (in kilograms) during adulthood, compared with weight maintenance (N=1758)

		**Absolute adult weight change between age 20 and middle age (in kilograms)**					
		**Model 1**		**Model 2**		**Model 3**	
	Proportion	**Ratio**	**95% CI**	**Ratio**	**95% CI**	**Ratio**	**95% CI**
HOMA-IR							
Loss of >5 kg	2.2%	1.08	0.84; 1.40	1.14	0.88; 1.50	0.95	0.72; 1.25
-5 kg - +5 kg (ref)	17.7%	1		1		1	
5 kg – 15 kg	42.3%	1.45	1.26; 1.67	1.46	1.27; 1.67	1.49	1.30; 1.71
15 kg – 30 kg	30.1%	2.03	1.78; 2.32	1.96	1.73; 2.23	2.04	1.80; 2.32
>30 kg	7.7%	3.13	2.69; 3.64	3.00	2.59; 3.47	3.12	2.70; 3.59
Matsuda ISI							
Loss of >5 kg	2.2%	1.04	0.83; 1.30	0.99	0.80; 1.23	1.16	0.92; 1.45
-5 kg - +5 kg (ref)	17.7%	1		1		1	
5 kg – 15 kg	42.3%	0.71	0.64; 0.79	0.71	0.64; 0.79	0.70	0.63; 0.77
15 kg – 30 kg	30.1%	0.52	0.47; 0.58	0.54	0.49; 0.60	0.53	0.48; 0.58
>30 kg	7.7%	0.35	0.31; 0.39	0.36	0.32; 0.41	0.35	0.31; 0.39

Results were based on analyses weighted towards the BMI distribution of the general population and were derived from beta coefficients with 95% confidence intervals from linear regression analyses and expressed as ratios of outcome measures compared with weight maintenance during adulthood. Abbreviations: CI, confidence interval; HOMA-IR, homeostatic model assessment insulin resistance; Matsuda ISI, Matsuda insulin sensitivity index; ref, reference. Model 1: adjusted for sex and age; 2: additionally adjusted for ethnicity, education, smoking, alcohol consumption, physical activity and family history of diabetes; 3: additionally adjusted for BMI at age 20.

Table S2. Association between of adult weight change (per 10% change) and measures of insulin resistance and sensitivity, for complete study population and for men and women separately

	**All** ***(N=1758)***		**Men** ***(n=913)***		**Women** ***(n=845)***	
	Beta	95% CI	Beta	95% CI	Beta	95% CI
**HOMA-IR**						
Model 1	1.21	1.18; 1.24	1.21	1.17; 1.26	1.21	1.16; 1.25
Model 2	1.20	1.17; 1.23	1.20	1.16; 1.24	1.20	1.15; 1.24

Model 3	1.25	1.21; 1.28	1.28	1.24; 1.33	1.22	1.18; 1.27
**Matsuda ISI**						
Model 1	0.83	0.81; 0.85	0.83	0.80; 0.86	0.83	0.81; 0.86
Model 2	0.84	0.82; 0.86	0.85	0.82; 0.87	0.84	0.82; 0.86
Model 3	0.81	0.80; 0.83	0.80	0.77; 0.82	0.83	0.80; 0.85

Results were based on analyses weighted towards the BMI distribution of the general population and were derived from beta coefficients with 95% confidence intervals from linear regression. Abbreviations: CI, confidence interval; HOMA-IR, homeostatic model assessment insulin resistance; Matsuda ISI, Matsuda insulin sensitivity index. Model 1: adjusted for sex and age; 2: additionally adjusted for ethnicity, education, smoking, alcohol consumption, physical activity and family history of diabetes; 3: additionally adjusted for BMI at age 20.

Table S3. P-values for interaction between total body fat, visceral adipose tissue and hepatic triglyceride content and adult weight change in the association with measures of insulin resistance (N=1758)

	**Total body fat**	**Visceral adipose tissue**	**Hepatic triglyceride content**
	P for interaction	P for interaction	P for interaction
Adult weight change			
HOMA-IR	0.247	0.684	0.458
Matsuda ISI	0.260	0.279	0.408

Results were based on analyses weighted towards the BMI distribution of the general population (N=1758), adjusted for sex, age, BMI at age 20, ethnicity, education, smoking, alcohol consumption, physical activity and family history of diabetes. Abbreviations: HOMA-IR, homeostatic model assessment insulin resistance; Matsuda ISI, Matsuda insulin sensitivity index.

Table S4. Mediation of the association between adult weight change and insulin resistance by total body fat, visceral adipose tissue and hepatic triglyceride content (N=1758)

	**Starting model (model 3)**		**+ TBF**		**+ VAT**		**+ TBF, VAT**		**+ HTGC**		**+ TBF, HTGC**		**+ TBF, VAT and HTGC**	
	**Ratio**	95% CI	**Ratio**	95% CI	**Ratio**	95% CI	**Ratio**	95% CI	**Ratio**	95% CI	**Ratio**	95% CI	**Ratio**	95% CI
HOMA-IR														

<-5.0%	0.73	0.47; 1.12	0.82	0.54; 1.25	0.83	0.55; 1.26	0.86	0.57; 1.31	0.76	0.49; 1.17	0.82	0.54; 1.25	0.85	0.56; 1.29
-5% - +5% (ref)	1		1		1		1		1		1		1	
+5% - 25%	1.47	1.30; 1.67	1.23	1.07; 1.42	1.13	0.99; 1.29	1.08	0.93; 1.24	1.29	1.14; 1.47	1.17	1.02; 1.35	1.07	0.93; 1.23
+25% - +50%	2.28	2.01; 2.59	1.58	1.31; 1.91	1.47	1.25; 1.73	1.32	1.09; 1.59	1.78	1.55; 2.06	1.45	1.21; 1.74	1.28	1.06; 1.54
>+50%	3.22	2.76; 3.77	1.87	1.48; 2.36	1.76	1.44; 2.16	1.49	1.17; 1.89	2.17	1.82; 2.58	1.59	1.27; 2.00	1.38	1.09; 1.74
Matsuda ISI														
≤5.0%	1.40	1.05; 1.86	1.25	0.94; 1.66	1.24	0.93; 1.66	1.19	0.89; 1.59	1.33	1.00; 1.77	1.25	0.94; 1.65	1.21	0.91; 1.61
-5% - +5% (ref)	1		1		1		1		1		1		1	
+5% - 25%	0.71	0.64; 0.79	0.84	0.75; 0.95	0.91	0.81; 1.02	0.95	0.85; 1.07	0.82	0.73; 0.91	0.89	0.80; 1.00	0.96	0.86; 1.08
+25% - +50%	0.47	0.42; 0.52	0.67	0.57; 0.78	0.70	0.61; 0.81	0.79	0.67; 0.92	0.61	0.54; 0.69	0.73	0.63; 0.86	0.81	0.69; 0.95
>+50%	0.34	0.30; 0.39	0.57	0.47; 0.70	0.59	0.50; 0.71	0.70	0.57; 0.87	0.52	0.44; 0.60	0.68	0.56; 0.83	0.77	0.63; 0.93

Results were based on analyses weighted towards the BMI distribution of the general population and were derived from beta coefficients with 95% confidence intervals from linear regression analyses and expressed as ratios of outcome measures compared with weight maintenance during adulthood. CI, confidence interval; HOMA-IR, homeostatic model assessment insulin resistance; Matsuda ISI, Matsuda insulin sensitivity index; TBF, total body fat; VAT, visceral adipose tissue; HTGC, hepatic triglyceride content; ref, reference. Model 3: adjusted for sex, age, BMI at age 20, ethnicity, education, smoking, alcohol consumption, physical activity and family history of diabetes. '+' indicates that the mediator(s) was/were added to the starting model (model 3).

Table S5. Mediation of the association between adult weight change and insulin resistance by total body fat, visceral adipose tissue and hepatic triglyceride content in men (n=913)

	**Starting model (model 3)**		**+ TBF**		**+ VAT**		**+ TBF, VAT**		**+ HTGC**		**+ TBF, HTGC**		**+ TBF, VAT and HTGC**	
	**Ratio**	95% CI	**Ratio**	95% CI	**Ratio**	95% CI	**Ratio**	95% CI	**Ratio**	95% CI	**Ratio**	95% CI	**Ratio**	95% CI
HOMA-IR														
<-5.0%	0.86	0.62; 1.18	1.13	0.85; 1.50	0.83	0.57; 1.20	1.02	0.74; 1.40	0.95	0.67; 1.36	1.15	0.84; 1.56	1.05	0.74; 1.50
-5% - +5% (ref)	1.00		1.00		1.00		1.00		1.00		1.00		1.00	
+5% - 25%	1.49	1.23; 1.80	1.16	0.94; 1.44	1.08	0.86; 1.34	0.97	0.77; 1.21	1.33	1.10; 1.62	1.14	0.93; 1.39	0.98	0.79; 1.22
+25% - +50%	2.36	1.95; 2.85	1.42	1.08; 1.88	1.47	1.14; 1.90	1.14	0.85; 1.53	1.86	1.50; 2.30	1.34	1.03; 1.74	1.13	0.86; 1.49
>+50%	3.70	2.84; 4.83	1.62	1.10; 2.38	1.98	1.41; 2.79	1.26	0.85; 1.88	2.54	1.91; 3.39	1.49	1.04; 2.11	1.23	0.85; 1.79
Matsuda ISI														
≤5.0%	1.41	0.99; 2.02	1.08	0.80; 1.47	1.45	0.96; 2.20	1.18	0.84; 1.67	1.26	0.84; 1.89	1.07	0.75; 1.51	1.14	0.78; 1.67
-5% - +5% (ref)	1.00		1.00		1.00		1.00		1.00		1.00		1.00	

+5% - 25%	0.73	0.61; 0.87	0.92	0.76; 1.12	0.97	0.78; 1.20	1.08	0.87; 1.33	0.82	0.68; 0.98	0.95	0.78; 1.14	1.06	0.87; 1.29
+25% - +50%	0.47	0.39; 0.56	0.76	0.59; 0.98	0.71	0.56; 0.90	0.92	0.70; 1.20	0.60	0.49; 0.73	0.81	0.64; 1.03	0.92	0.72; 1.19
>+50%	0.30	0.24; 0.39	0.67	0.47; 0.95	0.52	0.38; 0.72	0.82	0.57; 1.19	0.45	0.34; 0.58	0.73	0.53; 1.01	0.85	0.61; 1.18

Results were based on analyses weighted towards the BMI distribution of the general population and were derived from beta coefficients with 95% confidence intervals from linear regression analyses and expressed as ratios outcome measures compared with weight maintenance during adulthood. CI, confidence interval; HOMA-IR, homeostatic model assessment insulin resistance; Matsuda ISI, Matsuda insulin sensitivity index; TBF, total body fat; VAT, visceral adipose tissue; HTGC, hepatic triglyceride content; ref, reference. Model 3: adjusted for age and BMI at age 20, ethnicity, education, smoking, alcohol consumption, physical activity and family history of diabetes. '+' indicates that the mediator(s) was/were added to the starting model (model 3).

Table S6. Mediation of the association between adult weight change and insulin resistance by total body fat, visceral adipose tissue and hepatic triglyceride content in women (n=845)

	**Starting model (model 3)**		**+ TBF**		**+ VAT**		**+ TBF, VAT**		**+ HTGC**		**+ TBF, HTGC**		**+ TBF, VAT and HTGC**	
	**Ratio**	95% CI	**Ratio**	95% CI	**Ratio**	95% CI	**Ratio**	95% CI	**Ratio**	95% CI	**Ratio**	95% CI	**Ratio**	95% CI
HOMA-IR														
<-5.0%	0.67	0.39; 1.16	0.73	0.43; 1.24	0.81	0.48; 1.38	0.82	0.48; 1.39	0.68	0.39; 1.19	0.71	0.42; 1.22	0.78	0.46; 1.31
-5% - +5% (ref)	1.00		1.00		1.00		1.00		1.00		1.00		1.00	
+5% - 25%	1.43	1.20; 1.70	1.24	1.02; 1.50	1.14	0.96; 1.36	1.13	0.94; 1.36	1.23	1.03; 1.47	1.15	0.95; 1.39	1.09	0.91; 1.31
+25% - +50%	2.15	1.80; 2.55	1.58	1.22; 2.05	1.41	1.13; 1.76	1.38	1.06; 1.78	1.67	1.38; 2.02	1.43	1.11; 1.85	1.32	1.02; 1.71
>+50%	2.90	2.39; 3.52	1.89	1.39; 2.58	1.62	1.26; 2.09	1.56	1.15; 2.12	1.93	1.56; 2.39	1.57	1.16; 2.11	1.42	1.05; 1.91
Matsuda ISI														
≤5.0%	1.39	0.98; 1.98	1.30	0.91; 1.84	1.16	0.81; 1.66	1.16	0.81; 1.65	1.37	0.96; 1.96	1.32	0.93; 1.88	1.22	0.86; 1.73
-5% - +5% (ref)	1.00		1.00		1.00		1.00		1.00		1.00		1.00	
+5% - 25%	0.72	0.63; 0.81	0.82	0.71; 0.95	0.89	0.78; 1.01	0.90	0.78; 1.03	0.84	0.73; 0.95	0.89	0.77; 1.02	0.93	0.81; 1.07
+25% - +50%	0.48	0.42; 0.55	0.64	0.52; 0.79	0.72	0.60; 0.86	0.73	0.60; 0.91	0.63	0.54; 0.74	0.72	0.58; 0.88	0.77	0.63; 0.94
>+50%	0.37	0.31; 0.43	0.55	0.42; 0.71	0.64	0.52; 0.79	0.66	0.51; 0.85	0.57	0.47; 0.68	0.67	0.53; 0.86	0.74	0.58; 0.94

Results were based on analyses weighted towards the BMI distribution of the general population and were derived from beta coefficients with 95% confidence intervals from linear regression analyses and expressed as ratios of outcome measures compared with weight maintenance during adulthood. CI, confidence interval; HOMA-IR, homeostatic model assessment insulin resistance; Matsuda ISI, Matsuda insulin sensitivity index; TBF, total body fat; VAT, visceral adipose tissue; HTGC, hepatic triglyceride content; ref, reference. Model 3: adjusted for age and BMI at age 20, ethnicity, education, smoking, alcohol consumption, physical activity and family history of diabetes. '+' indicates that the mediator(s) was/were added to the starting model (model 3).
